# Mammographic density as a predictor of breast cancer survival: the Multiethnic Cohort

**DOI:** 10.1186/bcr3378

**Published:** 2013-01-22

**Authors:** Gertraud Maskarinec, Ian S Pagano, Melissa A Little, Shannon M Conroy, Song-Yi Park, Laurence N Kolonel

**Affiliations:** 1 University of Hawaii Cancer Center, 701 Ilalo Street, Honolulu, HI 96813, USA; 2 Canada Department of Population Health Research, Alberta Health Services-Cancer Care, Box ACB 2210-2nd Street SW, Calgary, AB, T2S 3C3, Canada

## Abstract

**Introduction:**

Mammographic density, a strong predictor for breast cancer incidence, may also worsen prognosis in women with breast cancer. This prospective analysis explored the effect of prediagnostic mammographic density among 607 breast cancer cases diagnosed within the Hawaii component of the Multiethnic Cohort (MEC).

**Methods:**

Female MEC participants, aged ≥ 50 years at cohort entry, diagnosed with primary invasive breast cancer, and enrolled in a mammographic density case-control study were part of this analysis. At cohort entry, anthropometric and demographic information was collected by questionnaire. Tumor characteristics and vital status were available through linkage with the Hawaii Tumor Registry. Multiple digitized prediagnostic mammograms were assessed for mammographic density using a computer-assisted method. Cox proportional hazards regression was applied to examine the effect of mammographic density on breast cancer survival while adjusting for relevant covariates.

**Results:**

Of the 607 cases, 125 were diagnosed as in situ, 380 as localized, and 100 as regional/distant stage. After a mean follow-up time of 12.9 years, 27 deaths from breast cancer and 100 deaths from other causes had occurred; 71 second breast cancer primaries were diagnosed. In an overall model, mammographic density was not associated with breast cancer-specific survival (HR = 0.95 per 10%; 95%CI: 0.79-1.15), but the interaction with radiotherapy was highly significant (p = 0.006). In stratified models, percent density was associated with a reduced risk of dying from breast cancer (HR = 0.77; 95%CI: 0.60-0.99; p = 0.04) in women who had received radiation, but with an elevated risk (HR = 1.46; 95% CI: 1.00-2.14; p = 0.05) in patients who had not received radiation. High breast density predicted a borderline increase in risk for a second primary (HR = 1.72; 95% CI: 0.88-2.55; p = 0.15).

**Conclusions:**

Assessing mammographic density in women with breast cancer may identify women with a poorer prognosis and provide them with radiotherapy to improve outcomes.

## Introduction

Mammographic density - the distribution of fat, connective, and epithelial tissue in the breast - has been used as a biomarker because a high percentage of dense parenchyma on mammographic images confers a four- to six-fold risk for breast cancer [1], but its relation to breast cancer survival has been much less studied. The histopathologic correlates of mammographic density are thought to represent dense connective tissues in addition to epithelial cells [2], but the amount of connective tissue is far greater (approximately 11-fold) than glandular tissue and contributes more to the variability in percentage of dense area [3-6]. It appears that the extracellular matrix (ECM) contributes to neoplastic progression and that disruptions in the ECM may precede epithelial changes [7]. Therefore, collagen-dense stroma associated with mammographic density may enhance tumor formation through epithelial-stromal interactions [8,9] and increase the likelihood for regrowth and progression [10]. Moreover, higher mammographic density may increase the probability of malignant initiation by representing a larger number of cells at risk that may proceed to neoplasia within the collagen-dense microenvironment [11].

So far, six reports have examined the influence of mammographic density on breast cancer outcomes: three on recurrence [12-14] and three on survival [15-17]. One study reported a higher risk of invasive breast cancer among ductal carcinoma *in situ *(DCIS) patients with highly dense breasts [12], and two investigations found higher local recurrence rates with higher density [13,14]. Three mammography cohorts using qualitative mammographic density assessment methods investigated breast density as a predictor of breast cancer-specific survival. An inverse association that was borderline significant was detected in a Swedish cohort [16], but no adverse effect of mammographic density on survival was found in a British [15] or a US [17] cohort. Based on the hypothesis that prediagnostic breast density adversely affects breast cancer outcomes, this analysis examined mammographic density as a predictor of breast cancer-specific survival after adjusting for important prognostic factors within the Hawaii component of the Multiethnic Cohort (MEC).

## Materials and methods

### Study population

A nested case-control study [18] within the MEC is the basis for the present analysis and was established in 1993-1996 to study diet and other exposures in relation to cancer among different ethnic groups in Hawaii and California [19]. The original cohort and the nested case-control study were approved by the Committee on Human Studies at the University of Hawaii. All subjects signed an informed consent form to participate in the study and a release form to allow the retrieval of mammograms. Subjects entered the cohort by completing a 26-page, self-administered mailed survey that asked about demographics, anthropometric measures, diet, lifestyle factors, and the presence of major co-morbidities (for example, diabetes, hypertension, and heart disease). Owing to logistic problems in retrieving mammograms in California, the mammographic density investigation was restricted to Hawaii [18]. For the nested case-control study, 1,587 potential cases were identified, 734 cases agreed to participate, and for 607 women mammograms could be obtained. Despite the relatively low participation rate, the included women were similar to the eligible subjects [18]. Linkage with the Hawaii Tumor Registry (HTR), part of the Surveillance, Epidemiology, and End Results (SEER) program, provided the most recent vital status, last date of contact, cause of death, and tumor characteristics, including stage at diagnosis and treatment during the first 6 months. Based on the primary cause of death, deaths were classified as those from breast cancer and those from other causes. Cancer recurrence information is not routinely collected by the HTR. Second breast cancers were identified according to SEER coding rules [20]; tumors in the contralateral breast and ipsilateral cancers diagnosed more than 5 years apart are considered multiple primaries.

### Mammographic density assessment

As described previously, multiple mammographic films were obtained for each woman to cover a wide time period; cases had a mean of 3.2 density measures [18]. The values for the right and the left breast and for the repeated density measures were averaged. The mean time between the earliest mammogram and the breast cancer diagnosis was 6.3 years, whereas the earliest and the latest mammogram were 5.1 years apart. Except for the film of five cases who only had a mammogram of the healthy breast taken at the time of diagnosis, all images were prediagnostic. The mammographic films from both breasts were scanned with a Kodak LS85 Film Digitizer (Eastman Kodak Company, Rochester, NY, USA) at a resolution of 98 pixels per inch. One of the authors (GM) performed computer-assisted density assessment by using Cumulus software [21] with excellent quality control results. In a random sample of 410 mammograms read in duplicate [18], the intraclass correlation coefficients were 0.96 (95% confidence interval (CI) 0.95 to 0.97) for the size of the dense areas and 0.974 for percent density (95% CI 0.968 to 0.978).

### Statistical analysis

We used the SAS package, release 9.3 (SAS Institute Inc., Cary, NC, USA) for all analyses. Follow-up time was calculated from the date of diagnosis until the earliest event of interest (that is, death, second breast cancer diagnosis, the last date of contact, or 1 August 2011). We used three approaches to examine percent density: as a continuous variable rescaled and expressed per 10% increase, as a dichotomous variable classified into low and high by using the median of 35%, and as three categories (less than 25%, 25 to less than 50%, or at least 50%). Body mass index (BMI) was categorized as normal, overweight, or obese. Because of the small number of events, hormone receptor status was classified as a two-level variable: (a) estrogen receptor-positive/progesterone receptor-positive (ER^+^/PR^+^), (b) ER^- ^or PR^-^, or unknown. Because it was not routinely performed before 2000, HER2/neu was not available.

Cox proportional hazard regression was applied to explore the relation of breast density with deaths due to breast cancer and other causes separately as well as with the incidence of a second breast cancer. Although mammographic density is unlikely to affect all-cause mortality, the analysis was added to show that breast density is not a marker for high mortality. When modeling second breast cancers, the time between first and second diagnosis was used as the follow-up time. Because a previous analysis in the MEC had shown that tumor stage, weight status, and ethnicity influenced survival, these variables were considered essential covariates [22]; all three changed the hazard ratio (HR) by more than 10%. Potential confounders - that is, menopausal status, co-morbidity (hypertension, diabetes, heart disease, or stroke), hormone replacement therapy, ER and PR status, and treatment (surgery, chemotherapy) - were evaluated. Because the results of the log-likelihood ratio test indicated no improvement in model fit (χ^2 ^= 6.43, 6 degrees of freedom (df), *P *= 0.38) and the HR for the overall model changed less than 10% (0.95 to 0.92), we decided not to include them. Given the previously reported effect modification by radiotherapy [13], we modeled the interaction between breast density and radiation by using cross-product terms and performed stratified analyses by radiation treatment. Owing to the limited sample size, separate analyses by ethnicity were not possible.

## Results

Japanese-Americans and Caucasians constituted the largest proportion of study participants (Table 1). Native Hawaiians, followed by Caucasians, had the highest BMI and the largest total breast area, whereas Japanese and 'Others' had the highest percent density because of their relatively small breast sizes. Of the 607 breast cancer cases, 125 were diagnosed as *in situ*, 380 as local, and 100 as advanced (regional/distant). After a mean follow-up time of 12.9 years, 27 deaths from breast cancer and 100 deaths from other causes occurred. By category, 215 women had less than 25%, 209 women had 25% to less than 50%, and 183 women had at least 50% breast density (Table 2). The respective values for the 25 breast cancer deaths were 9, 7, and 11 events.

**Table 1:** Characteristics of breast cancer cases by ethnic group

Characteristic	Native Hawaiian	Japanese	Caucasian	Other	All
Number of cases	79	292	195	41	607

Number of deaths	20	51	54	2	127

Breast cancer	7	10	9	1	27

All other causes	13	41	45	1	100

Stage at diagnosis, number					

*In situ*	10	68	38	9	125

Local	50	175	131	24	380

Advanced	19	48	25	8	100

Unknown	0	1	1	0	2

Number of second breast cancers	12	29	25	5	71

Mean age at mammograms, years	57.8	60.2	60.4	55.5	59.6

Age at diagnosis, years	61.7	63.9	63.9	59.3	63.3

Time from first mammogram to diagnosis, years^a^	6.3	6.7	6.7	6.4	6.3

Years of follow-up	12.4	13.2	12.6	13.8	12.9

Total breast area, cm^2^	139.4	92.0	136.7	98.0	112.9

Dense breast area, cm^2^	37.7	32.0	41.6	41.4	36.5

Percent density	30.1	38.2	36.2	45.1	37.0

Body mass index, kg/m^2^	28.2	24.3	25.2	24.4	25.1

Family history, percentage^b^	25.3	16.1	14.9	19.5	17.1

Age at menarche, years	12.9	12.9	13.0	13.8	13.0

Parous, percentage	87.3	84.3	82.1	80.5	83.7

At least 30 years old at first birth, percentage	3.8	10.3	8.7	14.6	9.2

Number of children	3.1	2.2	2.2	2.3	2.3

Postmenopausal, percentage	80.0	79.5	85.1	63.4	79.7

Use of any HRT, percentage^c^	48.1	24.3	34.9	31.7	31.3

ERT use, percentage	24.1	44.5	38.0	19.5	38.1

EPRT use, percentage	27.9	31.2	27.2	48.8	30.6

Values are presented as means unless indicated otherwise. ^a^Number of years from the earliest scanned mammogram to the diagnosis of breast cancer. ^b^Women who have first-degree relatives with breast cancer. ^c^Postmenopausal women are the denominator for the hormone replacement therapy (HRT) use. EPRT, combined estrogen-progestin replacement therapy; ERT, estrogen-only replacement therapy.

**Table 2:** Survival and incidence of new breast cancer related to breast density

Outcome	Model	Label	Per 10% density	Percent density^a^		Percent density categories		
				
				Low	High	< 25%	25 to < 50%	≥ 50%
Breast cancer death	All	Number	607	304	303	215	209	183
		
		Deaths	27	12	15	9	7	11
		
		HR^b^	0.95	1	0.83	1	0.60	0.89
		
		95% CI	0.79-1.15		0.36-1.92		0.22-1.66	0.34-2.33
		
		*P *value	0.61		0.67		0.33	0.80
	
	All plus interaction (density × radiation)	Number	607	304	303	215	209	183
		
		Deaths	27	12	15	9	7	11
		
		HR^b^	1.38	1	5.31	1	0.96	2.54
		
		95% CI	0.99-1.92		0.66-43.1		0.33-2.79	0.65-9.88
		
		*P *value	0.06		0.12		0.94	0.18
	
	With radiation	Number	361	175	170	128	131	102
		
		Deaths	16	11	5	9	2	5
		
		HR^b^	0.77	1	0.33	1	0.19	0.42
		
		95% CI	0.60-0.99		0.11-1.06		0.04-0.91	0.12-1.40
		
		*P *value	0.04		0.06		0.04	0.16
	
	Without radiation	Number	246	118	128	87	78	81
		
		Deaths	11	1	10	0	5	6
		
		HR	1.46	1	5.57		1	1.34
		
		95% CI	1.00-2.14		0.65-48.0			0.34-5.34
		
		*P *value	0.05		0.12			0.68

Other causes of death	All	Number	607	304	303	215	209	183
		
		Deaths	100	55	45	44	28	28
		
		HR^b^	1.08		1.37		1.06	1.32
		
		95% CI	0.98-1.20		0.88-2.13		0.64-1.75	0.77-2.25
		
		*P *value	0.13		0.16		0.83	0.32

Second breast cancer	All plus interaction (density × radiation)	Number	607	304	303	215	209	183
		
		Cases	71	32	39	22	23	26
		
		HR^b^	1.13	1	1.72	1	1.31	2.11
		
		95% CI	0.96-1.33		0.88-2.55		0.69-2.50	0.98-4.50
		
		*P *value	0.14		0.15		0.41	0.06

^a^Low density = < 35%; high density ≥ 35%. ^b^Hazard ratios (HRs) and 95% confidence intervals (CIs) for the effect of density estimated by Cox regression with adjustment for age at diagnosis, ethnicity, overweight/obesity, stage of disease at diagnosis (*in situ*, localized, or advanced), and radiation treatment when applicable.

The age at diagnosis of women with radiation was similar to that of women without radiation (63.0 versus 63.8 years; *P *= 0.28), and the stage distribution also did not differ (*P *= 0.43). In total, 361 women received radiation (Table 2), 57.6% of the *in situ*, 62.6% of local, and 59.0% of advanced cases. In those with radiation, 11 (5.9%) breast cancer deaths occurred in the low-density and 5 (2.9%) in the high-density group. Among the 246 women without radiation, 1 (0.9%) death was observed in the low-density group and 10 (7.8%) deaths in the high-density group.

After a mean time of 6.6 ± 4.4 years since the first diagnosis, 71 new breast cancer tumors were diagnosed: 56 occurred in the contralateral and 15 in the same side as the primary tumor. Among cases with secondary tumors, 38 women had received radiation and 33 had not.

**Figure 1:**
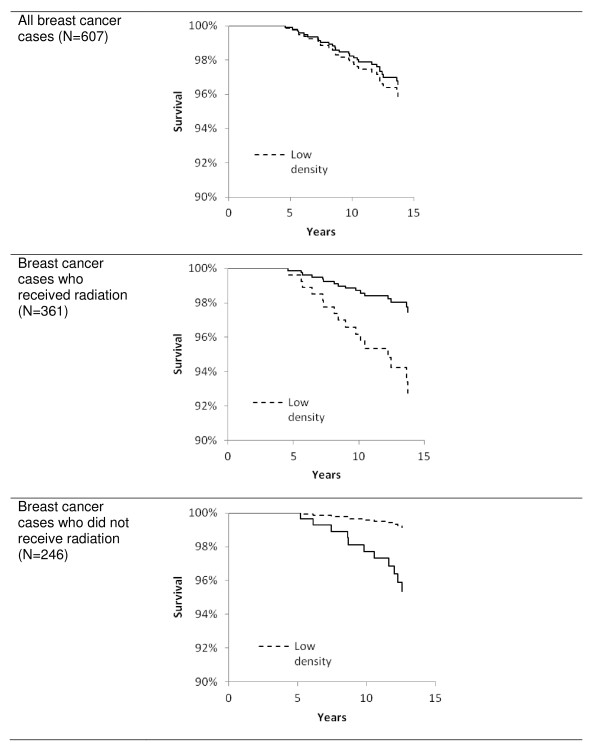
**Breast cancer survival stratified by radiation treatment in participants of a nested case-control study within the Multiethnic Cohort**. Survival by mammographic density (low density = less than 35%; high density = at least 35%) was estimated by Cox regression with adjustment for age at diagnosis, ethnicity, obesity, stage of disease at diagnosis (*in situ*, localized, or advanced), and radiation treatment.

In the overall model with 607 cases, mammographic density did not predict death due to breast cancer (Table 2). The HRs were 0.95 (95% CI 0.79 to 1.15) for percent density as a continuous variable and 0.83 (95% CI 0.36 to 1.92) as a dichotomous variable. Compared with HRs of women with less than 25% density, the HRs for the intermediate and high categories were 0.60 (95% CI 0.22 to 1.66) and 0.89 (95% CI 0.34 to 2.33), respectively. However, the interaction between percent density and radiation therapy was significant (*P *for continuous density = 0.006 and *P *= 0.02 for the two categorical analyses). At the same time, the risk estimates became elevated and borderline significant. The stratified models indicated divergent results for the two groups. Percent density was associated with a reduced risk of dying from breast cancer in women who had received radiation (HR = 0.77, 95% CI 0.60 to 0.99; *P *= 0.04) but with an elevated risk in patients who had not received radiation (HR = 1.46, 95% CI 1.00 to 2.14; *P *= 0.05). The results were similar when women were dichotomized by low and high density (Figure 1) and when three density categories were used (Table 2). Exclusion of *in situ *cases modified the HRs minimally because only one death occurred among them. The respective HRs for all breast cancer deaths with and without interaction using the continuous density variable were 0.92 (95% CI 0.76 to 1.12) and 1.29 (95% CI 0.91 to 1.81); the value for cases who had received radiation did not change and, for those without radiation, was 1.37 (95% CI 0.93 to 2.02).

Mammographic density was not associated with death from other causes when the continuous density variable (HR = 1.08, 95% CI 0.98 to 1.20) or density categories were used. However, the risk for a second breast cancer was 70% higher in women with high density (HR = 1.72, 95% CI 0.88 to 2.55; *P *= 0.15) and more than doubled when women with less than 25% were compared with those with at least 50% density (HR = 2.11, 95% CI 0.98 to 4.50). After exclusion of the 15 primaries that occurred in the ipsilateral breast, the risk estimates were attenuated. For example, the HRs for the intermediate- and high-density categories as compared with less than 25% were 1.15 (95% CI 0.45 to 2.97) and 1.43 (95% CI 0.51 to 4.01), respectively. No interaction with radiation was observed.

## Discussion

This investigation among 607 breast cancer cases with nearly 13 years of follow-up since diagnosis found an adverse effect of high mammographic density on breast cancer survival among patients who had not received radiation treatment. In contrast, among patients who had received radiation, women with dense breasts experienced a lower risk of death from breast cancer. Mammographic density was not associated with mortality from other causes of death. The non-significantly higher risk for a second primary breast cancer was attenuated after excluding ipsilateral cases, which are more likely to be recurrences. Also, radiotherapy did not modify the susceptibility of breast tissue to develop a second tumor.

The interaction seen here between breast density and radiation agrees with a report showing that women with high breast density had a significantly elevated risk to develop local breast cancer recurrence if they had not received radiotherapy [13]. Among 335 women, the respective proportions of recurrences for the low-, intermediate-, and high-density groups were 3 out of 99, 11 out of 107, and 20 out of 129. The 10-year actuarial risks were 21% versus 5% with an HR of 5.7 (*P *= 0.006) when the high group of patients was compared with the low-density group, but no association was seen in patients who received radiation, a finding that disagrees with the protective effect of radiation treatment on breast density seen in our study. In an investigation of 136 patients who had all received radiation, local recurrence rates were higher when the top quartile of breast density was compared with the lowest [14]. Women with 75% or more mammographic density experienced a 4.3-fold higher risk of local recurrence. Also, in agreement with our analysis, the risk of invasive breast cancer was threefold higher in women with very high breast density and a diagnosis of DCIS [12].

Despite the large number of cases, two mammography cohorts did not detect significant effects of mammographic density on breast cancer survival [15,17], whereas the Swedish cohort reported a borderline association of breast density with survival (*P *= 0.10) [16]. The density assessment by qualitative methods - that is, BI-RADS (Breast Imaging-Reporting and Data System) or Tabár - raises the question about the accuracy of the risk classification. In comparison with studies using quantitative methods, interobserver agreement is relatively low and data are often provided by multiple readers. Our findings also concur with an observation in the Nurses' Health Study, which found an association of more aggressive tumor characteristics (that is, size, ER-negative, and grade) in women with high breast density [23], and with a study in 231 British women who demonstrated a high probability for tumors to arise within parts of the breast that appear dense on mammographic images [24].

However, the previous studies disagree on whether radiotherapy modifies the association of mammographic density with recurrence [12-14]. Whereas one study observed an effect only in women without radiotherapy [13], another study whose participants had all received radiotherapy reported an increased risk [14], and a third study found that invasive breast cancer occurred after DCIS in women who received radiotherapy and in those who did not [12]. One of the studies found obesity to be a stronger predictor of local recurrence than breast density [14], but BMI was not included in the other study [13]. This may have introduced bias given that BMI is a negative confounder between breast density and breast cancer risk (that is, it has an inverse association with the former and a direct association with the latter) and is associated with poorer breast cancer survival [25]. None of the three mammography cohorts that examined breast cancer survival explored effect modification by radiotherapy [15-17].

The mechanisms underlying the paradoxical lower risk of mortality associated with the joint effects of high breast density and radiotherapy are unclear. Clinical trials have shown that chemotherapy and hormonal treatment have a higher impact on breast cancer survival but that treatment with breast-conserving surgery plus radiation has survival rates similar to those of mastectomy but higher local recurrence rates [26]. Therefore, our findings are more likely to be due to local disease recurrence and its after-effects [27] or a result of the so-called field effect (that is, the presence of cancer-like signatures in histologically normal tissues surrounding the primary tumor [28]). Adjuvant radiotherapy may favorably alter the tumor microenvironment and ECM to prevent recurrence [29-31]. It is possible that the collagen-dense stroma associated with mammographic density enhances the effectiveness of radiotherapy in preventing residual neoplastic cell growth and invasion. For example, the stromal matrix regulatory protein tissue metalloproteinase-3 (MMP-3) has been positively associated with mammographic density [5]; MMP-3 may play a critical role in the progression of residual neoplastic cells. Recently, MMP inhibitor-targeted treatment, in combination with radiotherapy, has been proposed as a strategy to improve breast cancer survival [32]. Furthermore, breast density has been associated with tumor growth factor-β activity [33], a potentially important mediator of the microenvironment's response to radiotherapy [29]. Unfortunately, we cannot determine whether the excess breast cancer mortality in cases without radiation was due to local or distant recurrence.

The major limitations of this study are the small number of deaths and the lack of recurrence information. The outcomes are based on data from a SEER registry, which does not routinely collect recurrence. However, the treatment data obtained directly from the HTR are considered of good quality because of the limited number of such facilities in the State of Hawaii and the low likelihood of receiving treatment out of state. Only 9 out of the 607 (1.5%) cases had a code indicating uncertainty about radiation treatment received. Strengths of our analysis are the availability of repeated mammograms, the long follow-up time, and the high quality of the mammographic density assessment method that provided quantitative estimates rather than qualitative categories.

## Conclusions

This longitudinal investigation with 13 years of follow-up found an adverse effect of high mammographic density on breast cancer survival among patients who had not received radiation treatment and a lower risk of death from breast cancer in women who had received radiation. To confirm the findings of this exploratory analysis, it appears feasible to reanalyze large breast cancer clinical trials for differences in outcomes according to breast density. Although chemotherapy was not significant in our study, effect modification with other treatment modalities could also be investigated, but there are biologic reasons to think that radiotherapy has a more pronounced effect on stroma, and, in particular, the ECMs that constitute mammographic density, than on breasts with a high proportion of fat tissue. Assessing mammographic density in women with breast cancer may help to identify women with a poorer prognosis. The implications of this research may be important for future treatment of breast cancer in women with high breast density who may benefit from additional radiotherapy.

## Abbreviations

BMI: body mass index; CI: confidence interval; DCIS: ductal carcinoma *in situ*; ECM: extracellular matrix; ER: estrogen receptor; HR: hazard ratio; HTR: Hawaii Tumor Registry; MEC: Multiethnic Cohort; MMP-3: metalloproteinase-3; PR: progesterone receptor; SEER: Surveillance, Epidemiology, and End Results.

## Competing interests

The authors declare that they have no competing interests.

## Authors' contributions

All authors made substantial contributions to conception and design, analysis and interpretation of data, and critical review of the manuscript. GM conceived of the study, coordinated the data acquisition and analysis, and helped to draft the manuscript. ISP participated in planning the statistical methods and performed part of the analysis. ML helped to draft the manuscript. SMC contributed to the data analysis and drafted parts of the manuscript. LNK and SYP participated in the design of the study and in the statistical analysis. All authors read and approved the final manuscript.
